# The clinical outcomes of mechanical thrombectomy for proximal M1 occlusion involving lenticulostriate perforators: Is it worse than distal M1 occlusion? Retrospective observational study

**DOI:** 10.1097/MD.0000000000027036

**Published:** 2021-08-27

**Authors:** Hak Cheol Ko

**Affiliations:** a Department of Neurosurgery, Stroke and Neurological Disorders Centre, Kyung Hee University Hospital at Gangdong, Seoul, South Korea; b College of Medicine, Kyung Hee University, Seoul, South Korea.

**Keywords:** functional outcome, lenticulostriate artery, mechanical thrombectomy, middle cerebral artery occlusion

## Abstract

Although the success rate of recanalization in acute intracranial artery occlusion is high, there is a poor rate of improvement in functional clinical outcome. The purpose of this study was to assess the functional outcome of mechanical thrombectomy for proximal M1 occlusion involving lenticulostriate arteries (LSAs) compared with distal M1 occlusion-sparing the LSAs.

A retrospective analysis was conducted in patients with middle cerebral artery (MCA) M1 occlusions who had a successful recanalization subsequent to mechanical thrombectomy. The recanalization results were estimated using the thrombolysis in cerebral infarction grade assessed by digital subtraction angiography. To confirm the ischemic change resulting from the lenticulostriate artery occlusion, we reviewed the neuroimaging findings from magnetic resonance imaging 1 day after mechanical thrombectomy. The functional outcomes were then evaluated using the modified Rankin scale at 90 days.

In total, 28 patients with MCA M1 occlusion had successful recanalization outcomes with thrombolysis in cerebral infarction grades IIa, IIb, and III. Among the 28 patients, 17 had proximal M1 occlusions and 11 had distal M1 occlusions. Demographic factors, including initial National Institutes of Health Stroke Scale score, time from symptom to recanalization, and recanalization rate did not differ considerably between patients with proximal and distal M1 occlusions. Regarding infarctions in the basal ganglia, internal capsule, and corona radiata, there were statistically significant differences between the proximal and distal M1 occlusions. However, there were no significant differences in good functional outcome (modified Rankin scale ≤2) observed between the groups at 90 days after mechanical thrombectomy.

Although proximal M1 occlusion had more frequent infarctions associated with the LSA territories, these were not related to poor functional outcomes. Both proximal and distal M1 occlusion demonstrated comparably good outcomes.

## 1 Introduction

Acute middle cerebral artery (MCA) occlusion is a critical condition known to comprise 49.7% of all intracranial artery occlusion cases.^[1]^ If left untreated, MCA occlusions can cause catastrophic neurological deterioration, greatly impacting an individual's quality of life. However, in recent years, endovascular recanalization for an acute occlusion of a major intracranial artery by means of a stent retriever device has remarkably advanced, thus reducing the associated disastrous consequences to a significant degree.

Although great progress has been made in increasing the technical success rate of recanalization, this has not led to an equivalent improvement in functional clinical outcome. The factors influencing the functional outcome include the timing, the patient's age, the patients score on the National Institute of Health Stroke Scale (NIHSS) upon admission, the patient's systemic condition (e.g., systolic blood pressure, hyperglycemia, etc), and numerous vasculature factors. The vasculature factors are particularly important and include the extent of early ischemic changes before recanalization, the status of the collateral circulation which affects the degree of perfusion status and determines the infarct core and penumbra, the degree of recanalization, and the exact location of the occlusion.

Various approaches have been used for predicting the outcome of patients with acute occlusion of a major intracranial artery following endovascular recanalization. Among them, thrombolysis in cerebral infarction (TICI), which Higashida et al^[2]^ proposed for assessing the angiographic recanalization of acute occlusion of the intracranial artery has been widely used in the literature either in its original or modified form. Based on the TICI scale, many clinical trials have attempted to establish more effective and safer guidelines as well as to estimate the outcome of acute ischemic stroke patients.

However, there is a lack of documented reports on functional outcomes after recanalization among the different types of artery occlusions, especially MCA. Therefore, this study was designed to assess the functional outcomes of recanalization after M1 segment occlusion, which subdivides into the proximal half of the M1 (involving the lenticulostriate vessels) and the distal half of the M1 (sparing the lenticulostriate vessels).

## 2 Materials and methods

We retrospectively reviewed our database of mechanical thrombectomy for acute occlusion of the intracranial artery from December 2010 to February 2016. Three reviewers independently extracted the clinical data from the electronic records including initial clinical status, patient demographics, thrombectomy procedures, radiological findings, treatment results, and clinical outcome. The radiological findings were confirmed by 2 experienced neuroradiologists. The NIHSS and modified Rankin scale (mRS) were used to assess clinical status.

### 2.1 Inclusion and exclusion criteria

Patients were selected as follows: time window within 6 hours from symptom onset to start of procedure of mechanical thrombectomy; M1 segment occlusion of MCA; only a stent retriever was used for the thrombectomy; brain computed tomography angiogram (CTA) was performed before the thrombectomy procedure; brain magnetic resonance imaging including MR diffusion weighted images (DWI) were checked 24 hours after the procedure; and the clinical outcome was measured with mRS at 90 days post procedure.

The exclusion criteria included: patients with a tandem occlusion of the internal carotid artery (ICA) and terminal ICA; patients with underlying severe stenosis of M1; and patients with an unsatisfactory recanalization (TICI score of 0 and 1).

### 2.2 Parameters of assessment

**Figure 1:**
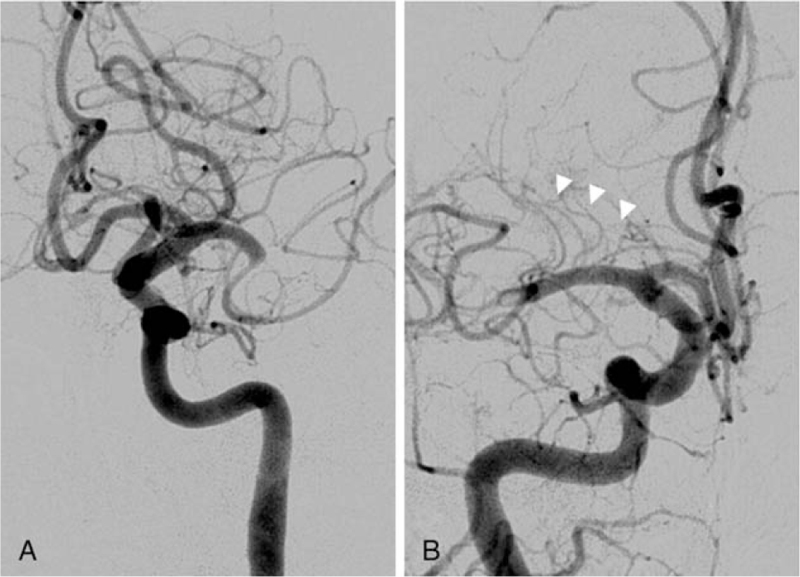
Digital subtraction angiography of M1 occlusion. A, The proximal M1 occlusion involving the lenticulostriate arteries that were not visualized. B, The distal M1 occlusion sparing the lenticulostriate arteries (white arrowheads).

**Figure 2:**
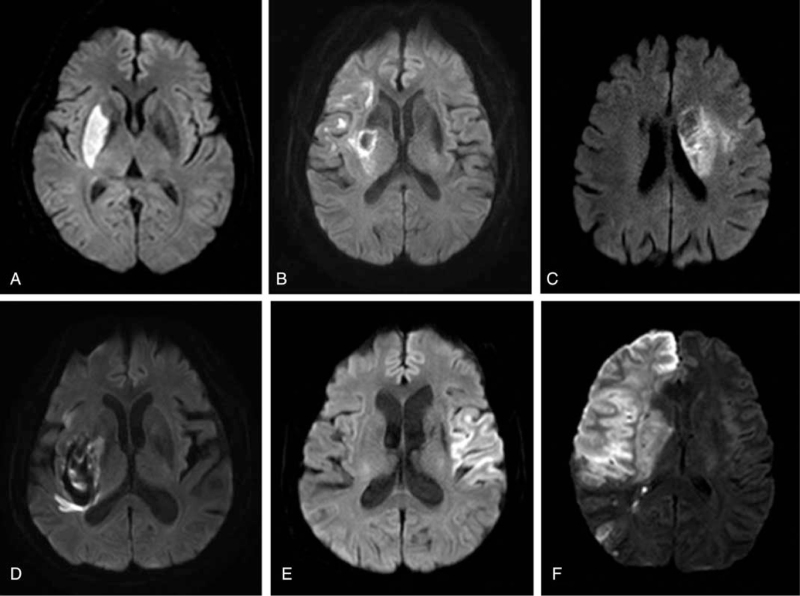
Examples of acute ischemic stroke from each specific location on magnetic resonance diffusion-weighted images. A, Basal ganglia infarction. B, Internal capsule infarction. C, Corona radiata infarction. D, Hemorrhagic transformation of basal ganglia infarction. E, Territory infarction of M2 superior division. F, Territory infarction of total middle cerebral artery.

The distal and proximal M1 occlusions were defined according to whether they spared the lenticulostriate arteries (LSAs) or not. The proximal M1 occlusions involved the LSAs that were not visualized on digital subtraction angiography, while the distal M1 occlusions spared the LSAs (Fig. 1). The assessment of collateral flow was measured using a preprocedural CTA. We used the grading system of collateral status that was proposed by Tan et al^[3]^ as follows: grade 0 = absent collateral supply to the occluded MCA territory; grade 1 = collateral supply filling <50% but >0% of the occluded MCA territory; grade 2 = supply filling >50% but <100% of the MCA territory; and grade 3 = 100% collateral supply of the occluded MCA territory. All CTA images were 7-mm maximum intensity projection reconstructions and 4-mm axial reformats. We used the same CTA imaging technique and divided the grading system as poor collaterals (grades 0 and 1) and good collaterals (grades 2 and 3). We confirmed irreversible acute stroke after 24 hours on control MR DWI. Basal ganglia (BG), internal capsule (IC), corona radiata (CR), territory infarctions, and BG hemorrhagic transformation were regarded as key parameters that could result in moderate to severe disability. Figure 2 shows examples of acute ischemic stroke for each specific location on MR DWI. The TICI perfusion scale was used for angiographic recanalization, and clinical outcome was assessed by mRS at 90 days. Good functional outcome was defined as an mRS score of 0 to 2.

Other outcome predictors included: demographic factors (age and sex), underlying diseases (hypertension, diabetes mellitus, dyslipidemia, atrial fibrillation, heart valve disease, and cardiomyopathy), onset to recanalization time, and initial NIHSS score.

### 2.3 Mechanical thrombectomy procedure

All procedures were performed under local anesthesia. Mechanical thrombectomy in this study was carried out using stentretriever—Solitaire AB (ev3 Neurovascular, CA), Solitaire FR (ev3 Neurovascular, Irvine, CA), Trevo XP (Stryker Neurovascular, Fremont, CA), and Trevo ProVue (Stryker Neurovascular, Fremont, CA). Proximal suction aspiration through a guide catheter was performed, a balloon guide catheter was used since November 2014.

### 2.4 Statistical analysis

Statistical analysis was performed using commercially available software (SPSS version 18.0, SPSS Inc., Chicago, IL). As a nonparametric test, the Mann–Whitney *U* test was used for the continuous parameters, including age, onset to recanalization time, and NIHSS score. A Student *t* test was used to compare the remaining non-continuity variables. A *P* value of <.05 was considered significant.

## 3 Results

In total, 28 patients with pure M1 occlusions and successful recanalization after mechanical thrombectomy met the inclusion and exclusion criteria in this study. Demographic data are summarized in Table 1. The demographic characteristics and underlying diseases were not significantly different between the 2 subgroups of proximal and distal M1 occlusion. The initial NIHSS score, intravenous administration of tissue plasminogen activator, and onset to recanalization time were also comparable. Table 2 shows the clinical and radiological results of mechanical thrombectomy in the proximal M1 and distal M1 occlusion groups. The recanalization results represented by the TICI grade and good collaterals on CTA did not differ significantly between the proximal M1 and distal M1 groups. Although BG, IC, and CR infarctions in 24 hours control MR DWI were detected significantly more frequently in the patients with a proximal M1 occlusion, there was no significant difference in the functional outcome at 90 days after thrombectomy between the 2 groups (Good outcome of mRS ≤2 of proximal M1/distal M1 = 47%/63%, *P* = .320).

**Table 1:** Demographic characteristics of patients with proximal M1 and distal M1 occlusions.

Characteristics	Total (n = 28)	Proximal M1 (n = 17)	Distal M1 (n = 11)	Significance (*P*)
Sex (M/F, n)	16/12	9/8	7/4	.705
Age (mean ± SD, yrs)	70.79 ± 8.88	70.88 ± 9.64	70.64 ± 8.00	.851
Initial NIHSS (mean ± SD)	14.82 ± 5.28	15.29 ± 4.99	14.09 ± 5.86	.619
o/s to recanalization time (mean ± SD, min)	299.29 ± 62.16	290.88 ± 51.43	312.27 ± 76.79	.378
IV tPA (n)	16	10	6	.565
Underlying diseases				
A fib (n)	20	14	6	.070
Heart valve disease /cardiomyopathy (n)	3	2	1	.664
HTN (n)	18	12	6	.444
DM (n)	7	2	5	.076
Dyslipidemia (n)	10	5	5	.444

A fib = atrial fibrillation, DM = diabetes mellitus, F = female, HTN = hypertension, IV = intravenous, M = male, NIHSS = National Institutes of Health Stroke Scale, o/s = onset, SD = standard deviation, tPA = tissue plasminogen activator.

**Table 2:** Clinical and radiological results of mechanical thrombectomy of proximal M1 and distal M1 occlusion.

	Total (n = 28)	Proximal M1 (n = 17)	Distal M1 (n = 11)	Significance (*P*)
TICI grade (n)				.889
2a	10	6	4	
2b	9	6	3	
3	9	5	4	
Good collaterals on initial CTA (n)	8	5	3	.624
MR DWI within 24 hrs after thrombectomy				
BG infarction (n)	20	16	4	.002
IC infarction (n)	5	5	0	.063
CR infarction (n)	11	11	0	.001
Territory infarction (n)	12	6	6	.269
Hemorrhagic transformation on BG (n)	7	6	1	.131
Good outcome of mRS ≤2 (n)	15	8	7	.320

BG = basal ganglia, CR = corona radiata, CTA = computed tomography angiogram, DWI = diffusion-weighted image, IC = internal capsule, MR = magnetic resonance, mRS = modified Rankin scale, TICI = thrombolysis in cerebral infarction.

We also analyzed the predictors that influenced a good clinical outcome at 90 days after treatment and found that there were no meaningful variables among the demographic factors, initial clinical status, recanalization time, and underlying diseases. Contrary to this expectation, the location of the occlusion site of M1 (proximal vs distal) and BG infarction on 24 hours control MR DWI were not predictors of functional outcome. Instead, the strongest predictors of outcome were IC, CR, and territory infarctions (Table 3).

**Table 3:** Predictors of good clinical outcome with modified Rankin scale ≤2.

	mRS ≤ 2 (n = 15)	mRS > 2 (n = 13)	Significance (*P*)
Sex (M/F, n)	10/5	6/7	.239
Age (mean ± SD, yrs)	69.67 ± 9.27	72.08 ± 8.58	.344
Initial NIHSS (mean ± SD)	14.20 ± 6.14	15.34 ± 4.19	.532
o/s to recanalization time (mean ± SD, min)	299.00 ± 66.93	299.62 ± 58.89	.908
IV tPA (n)	9	7	.521
Underlying diseases			
Afib (n)	10	10	.431
Heart valve disease /cardiomyopathy (n)	2	1	.566
HTN (n)	7	11	.055
DM (n)	5	2	.258
Dyslipidemia (n)	5	5	.544
Location of occlusion (n)			.320
Proximal M1	8	9	
Distal M1	7	4	
IV tPA (n)	9	7	.521
TICI grade (n)			.113
2a	3	7	
2b	7	2	
3	5	4	
Good collaterals on initial CTA (n)	6	2	.155
MR DWI within 24 hrs after thrombectomy			
BG infarction (n)	10	10	.431
IC infarction (n)	0	5	.013
CR infarction (n)	3	8	.031
Territory infarction (n)	3	9	.012
Hemorrhagic transformation on BG (n)	2	5	.137

A fib = atrial fibrillation, BG = basal ganglia, CR = corona radiata, CTA = computed tomography angiogram, DM = diabetes mellitus, DWI = diffusion-weighted image, F = female, HTN = hypertension, IC = internal capsule, IV = intravenous, M = male, MR = magnetic resonance, mRS = modified Rankin scale, NIHSS = National Institutes of Health Stroke Scale, o/s = onset, SD = standard deviation, TICI = thrombolysis in cerebral infarction, tPA = tissue plasminogen activator.

## 4 Discussion

Since the introduction of intra-arterial recanalization using either thrombolytic agents or mechanical thrombectomy, the most predictive factors for determining patients’ outcomes have been reported to be the initial NIHSS, the status of the collateral circulation, the success rate of recanalization, location of occlusion, infarct volume, and hemorrhagic transformation.^[4–9]^ Among these important factors, the location of the occlusion and whether it is possible to recanalize without complication have remained the factors that physicians are most concerned about. Given that acute MCA occlusions are among the most common type of occlusions, it is worthwhile to analyze them in detail based upon their anatomical subdivision (i.e., proximal M1 and distal M1 occlusion).

The present study demonstrates that functional outcomes after recanalization of M1 occlusions are not different based upon their location (proximal M1 vs distal M1). The crucial factors that are important in determining the functional outcome in MCA occlusion seem to be the patency of LSAs emerging from the M1 segment of the MCA. The LSAs do not communicate with other perforators, known as “end arteries,”^[10–12]^ and are subdivided into medial LSAs, which are anterior to the cerebral artery, and LSAs which are lateral from the MCA. The lateral LSAs supply the posterolateral head of caudate nucleus, the anterior portion of the body of caudate nucleus, tail of caudate nucleus, anterior putamen, and external part of the globus pallidus.^[12,13]^ Nevertheless, the globus pallidus, the lower part of the genu as well as the posterior limb of the IC, and the posteromedial portion of the lentiform nucleus are generally supplied by the anterior choroidal artery, the recurrent artery of Heubner, and perforators from the posterior communicating artery. A study by Behme et al^[14]^ found that proximal M1 occlusions showed an unfavorable clinical outcome in comparison with distal M1 occlusions, which was attributed to IC infarction. Obviously, the IC is an important location in connecting the pyramidal tract, but a proximal M1 occlusion (i.e., occlusion of the lateral LSAs) is not likely to incapacitate the entire IC because the basal IC genu and posterior limb of the IC are usually supplied through the anterior choroidal artery and the posterior communicating artery. In addition, Kleine et al^[15]^ reported that, in lateral LSAs occlusions, the IC involvement is much less consistent than involvement of the striatum due to the greater ischemic tolerance of white matter compared with gray matter.

In this study, there were no significant differences in initial NIHSS, the status of the collateral circulation, the success rate of recanalization, and hemorrhagic transformation between proximal and distal M1 occlusion patients. However, BG and CR infarctions occurred more frequently in proximal M1 occlusions than in distal M1 occlusions, which is in accordance with lateral LSAs occlusion. Nevertheless, the final functional outcomes of proximal M1 occlusions were similar to those of distal M1 occlusions because the ICs were spared in both proximal and distal M1 occlusion. Several studies have found results similar to ours. One prior study reported that there was no difference in the Alberta Stroke Program Early CT score and low density of IC on CT after mechanical thrombectomy for proximal M1 and distal M1 occlusion.^[16]^ These results may be explained by the fact that baseline ischemic core had a greater effect on the clinical outcome than the occlusion site after mechanical thrombectomy.^[17]^ Another study reported no difference in the clinical outcome between proximal M1 and distal M1 occlusion, which could explain why thrombus first causes ischemic damage to the basal ganglia by obstructing the orifice of perforators originating from the mid-M1, then spontaneously migrating to the distal MCA causing distal M1 occlusion.^[18]^

Our study was intended to help physicians facing acute MCA occlusions to provide an estimation of their functional outcome along with M1 segmental subdivision. Importantly, both proximal and distal M1 occlusions demonstrated comparably favorable functional outcomes in terms of IC preservation.

The final good functional outcomes were not significantly different between proximal M1 and distal M1 occlusions (47% vs 63%, *P* = .320) in our study. Although the etiology of acute stroke has not previously been investigated between proximal M1 and distal M1 occlusions, Kim et al^[16]^ reported that proximal M1 occlusions were more likely to be caused by atherosclerosis, whereas distal M1 occlusions were more likely to be caused by cardio-embolism, and that a good clinical outcome (mRS ≤ 2) was achieved for atherosclerotic occlusion and cardioembolic occlusion. The clinical outcomes of atherosclerotic occlusion may be poor because of the possibility of reocclusion after mechanical thrombectomy; however, according to a previous report, with rescue management, clinical outcomes can be improved by reducing the rate of reocclusion in atherosclerotic occlusion.^[19]^ Another previous study has reported that there is no evidence of significant interaction between ischemic core and occlusion location.^[17]^ That is, the baseline ischemic core was shown to be a more powerful predictor of functional outcome than the occlusion location, but the relationship between ischemic core and outcome does not different by occlusion locations.^[17]^ Although acute ischemic stroke with a distal M1 occlusion have higher rate of favorable outcome than a proximal M1 occlusion, the baseline ischemic core, that is influenced by the physiology such as collateral status, vessel patency status, has a greater influence on functional outcome than the occlusion location associated with LSAs involvement.

Although, in this study, the differences in clinical outcomes according to the thrombectomy approach (contact aspiration vs stent retriever) were not compared, Lapergue et al^[20]^ previously reported that there was no significant difference in the total number of revascularization attempts, median time from arterial puncture to revascularization, early improvement in neurological outcomes, or final functional outcomes between the contact aspiration technique and stent retriever technique. However, to obtain a better rate of recanalization, combining contact aspiration and stent retriever may be necessary. A previous study has found no significant relationship between the distance-to-thrombus from ICA bifurcation and the 90-days mRS, although successful recanalization rate was more frequent in shorter thrombus distance from ICA bifurcation.^[21]^ It may be influenced by the thrombectomy approach and the variation of the origin of LSAs.

This preliminary study has some limitations. First, the population of patients was relatively small. However, we placed emphasis on analyzing single-vessel occlusions among all intracranial vessel occlusions. Thus, this report might still be sufficient for displaying functional outcomes between proximal M1 and distal M1 occlusions. Second, this study was retrospectively investigated, and data collection through medical records can increase the risk of selection bias. Therefore, studies incorporating larger sample sizes and a prospective study design are needed to resolve these limitations.

## 5 Conclusions

Although proximal M1 occlusions had more frequent infarctions associated with the LSAS territories, they were not found to have a poorer functional outcome than distal M1 occlusions. In fact, we were able to present comparably favorable outcomes for both proximal and distal M1 occlusions. Our study may reconfirm the importance of an initial NIHSS assessment, the time to recanalization from symptom onset, the status of collateral circulation, the success rate of recanalization, and the infarction volume after recanalization.

## Acknowledgments

The authors would like to thank the patient who consented to this report and Editage (www.editage.com) for English language editing.

## Author contributions

**Conceptualization:** Hak Cheol Ko.

**Data curation:** Hak Cheol Ko.

**Investigation:** Hak Cheol Ko.

**Writing – original draft:** Hak Cheol Ko.

**Writing – review & editing:** Hak Cheol Ko.
